# Supplementary material for: Wireless in-body sensing through genetically engineered bacteria
Source: Nat Commun. 2025 Nov 25;16:10432. doi: 10.1038/s41467-025-65416-5 (PMC12647575; doi:10.1038/s41467-025-65416-5)
Supplement: Supplementary file 1 — Supplementary Information [file 41467_2025_65416_MOESM1_ESM.pdf]

Supplementary Information

Table S1: Primers utilized to amplify the target region for responsible cloning (sequence from 5' to 3')

P1	CAAATAATTTTGTTTAACTTTAAAGAGGAGAAAGGTACCATGGGTATGCTTGAAGCC
P2	GAGCCTTTCGTTTTATTTGATGCCACGCGTTTATTTACTCTCCTGCGGCG
P3	AAAAAAGGTACCTTTCTCCTCTTTCTCTAGTAGCTAGC
P4	AAGCTTGGATCCCTGCAG

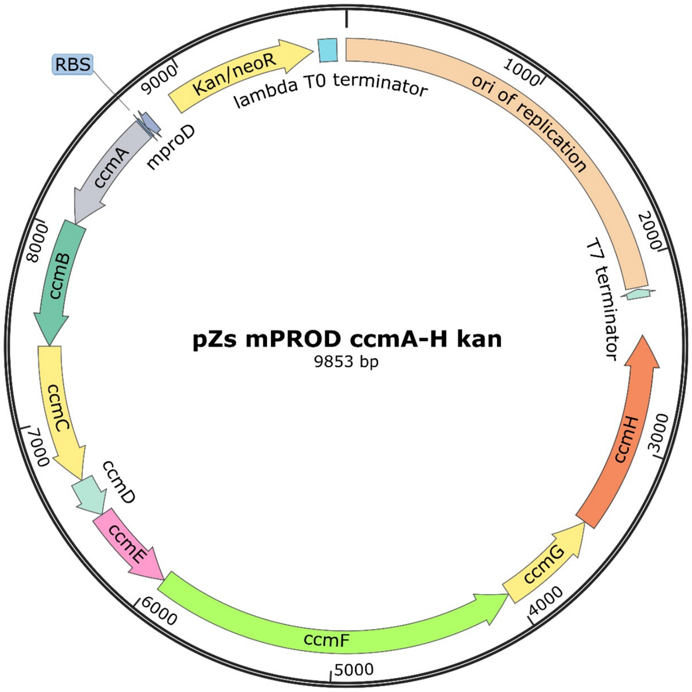

Figure S1: Representation of plasmid map for pZs mPROD ccmA-H kan cloning.

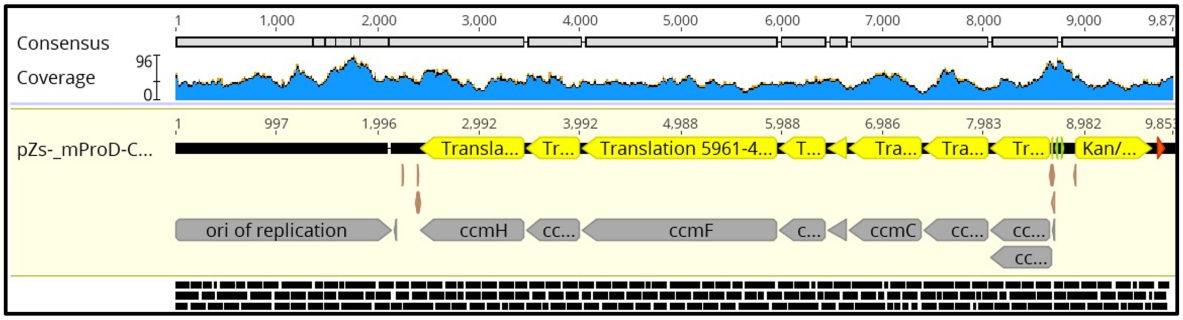

Figure S2: Next-generation sequencing (NGS) verification result for pZs-mProD-ccmA-H kan cloning.

EM Simulation Results

The simulation model is shown in Figure 9 of the manuscript. Simulations were performed both with and without the implant antenna, and the transmission coefficient was calibrated using the method described in the manuscript. The resulting data are presented in Figure S3. Consistent with the

measurement results shown in Figure 10, the implant antenna exhibits a resonant frequency near 1.2 GHz.

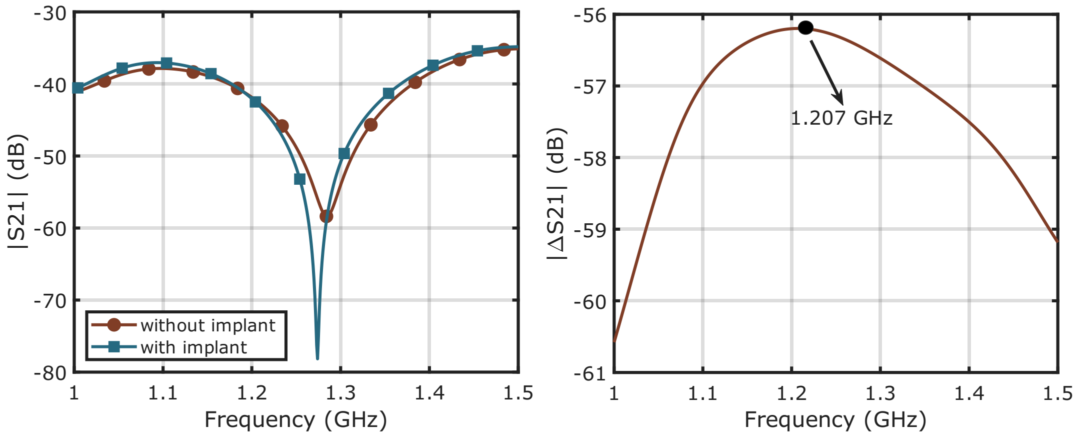

Figure S3: The simulated transmission coefficients. $|S_{21}|$ of the on-body antenna with and without implant (left) and the calibrated $|S_{21}|$ (right).

EM Measurement Results

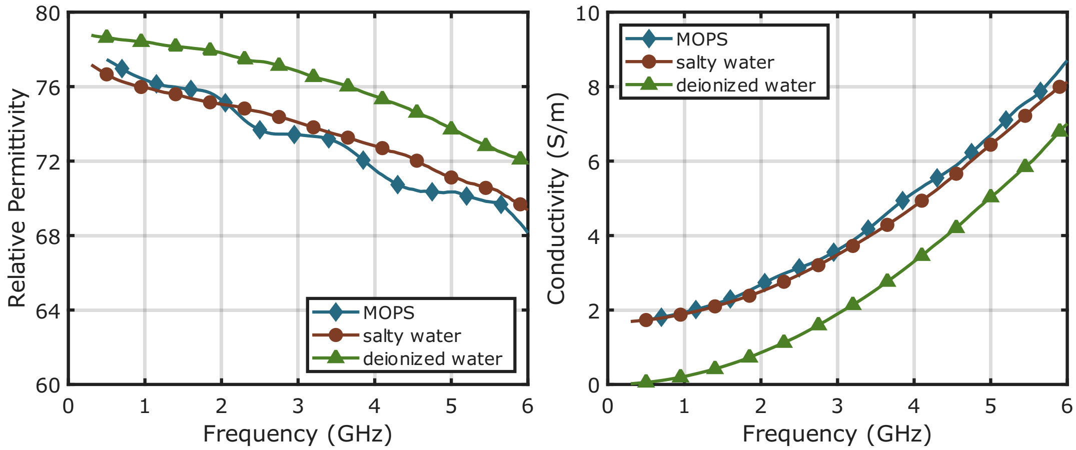

Figure S4: Frequency-dependent electrical properties of MOPS, MOPS-mimicking phantom (salty water), and deionized water.

Measurements were conducted using the same setup shown in Figure 9. To replicate the electrical properties of MOPS, a MOPS-mimicking phantom was prepared using a salt-water solution, as illustrated in Figure S4. Additionally, a water–glycerol mixture was used to mimic muscle tissue, and the effect of glycerol concentration on the mixture's electrical properties is shown in Figure S5.

To investigate the reliable sensing depth, an implant antenna is prototyped on an RO3003 substrate with a thickness of 1.57 mm, as shown in Figure S6 (a).

The measurement procedure involved three steps: first, the S-parameters were recorded without the implant present in the setup ($S_{xy,1}$). Next, the implant antenna was placed inside a 3D-printed cup, and a second set of S-parameters was recorded ($S_{xy,2}$). Finally, the antenna was removed, and a third set of S-parameters was measured ($S_{xy,3}$). This procedure was repeated for implant depths ranging from 25 mm to 65 mm, in 10 mm increments.

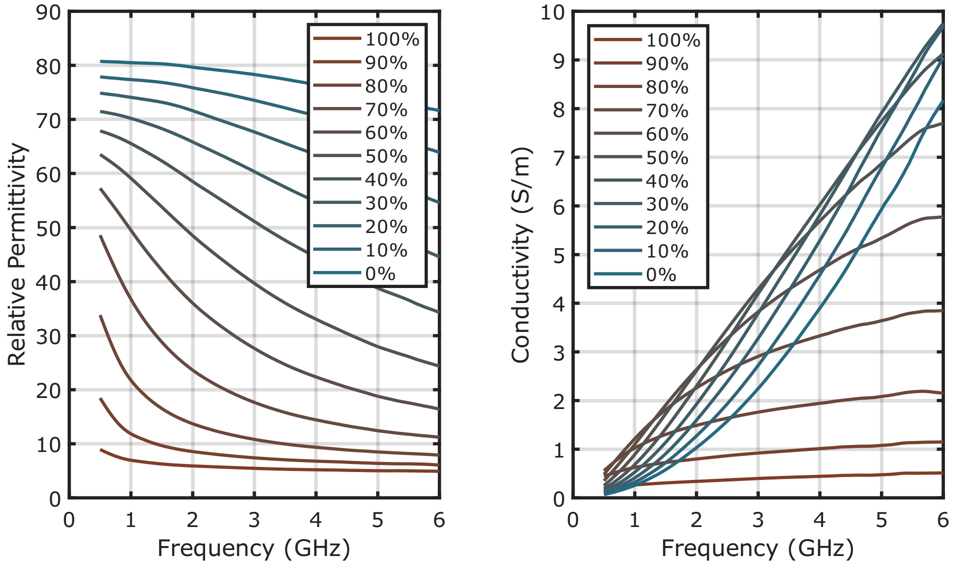

Figure S5: Frequency-dependent electrical properties of various glycerol-water solutions. The percentages in the legend indicate the volume ratio of glycerol to the total solution volume.

For each measurement, the same calibration method described in the manuscript was applied. The initial measurement ($S_{21,1}$) served as the reference for calibration. The calibrated transmission coefficient in the final measurement without the antenna, defined as $|\Delta S_{21,3}| = |S_{21,3} - S_{21,1}|$, represents the noise level, since both cases lacked the implant. The calibrated transmission coefficient when the implant was present, $|\Delta S_{21,2}| = |S_{21,2} - S_{21,1}|$, reveals the resonant frequency of the implant antenna.

The results are shown in Figure S6 (b-f). As the implant depth increases, the backscattered signal from the implant antenna becomes weaker. At 55 mm depth, the resonance is still discernible, while at 65 mm, it is no longer visible.

Note that since the antenna is non-biodegradable and fabricated on a commercial substrate to simplify the measurement process, its resonant frequency differs slightly from that of the biodegradable version.

To demonstrate the effect of tissue conductivity on the resonance quality, the calibrated transmission coefficients are presented in Figure S7, where the implant depth is fixed at 35 mm. The measurement was repeated using deionized water instead of the MOPS-mimicking phantom. As shown in Figure S4, deionized water has significantly lower conductivity compared to the MOPS-mimicking phantom. Due to the reduced conductivity of the surrounding medium, the implant antenna exhibits a higher quality factor, resulting in a sharper and more pronounced resonance.

Detuning of the implant resonance may occur due to changes in the permittivity of the surrounding medium. Since the implant antenna is directly exposed to the bacterial medium, its effective permittivity is initially close to that of water ($\sim$80), as shown in Figure S4 (left). To quantify the effect of detuning, we performed additional waveguide simulations by varying the relative permittivity of the surrounding medium from 80 down to 20, as seen in Figure S8.

- At $\epsilon_r = 80$ (MOPS-like medium), the resonance appears around 1.15 GHz.
- As ϵ_r decreases to 20, the resonance shifts upward to 1.75 GHz.

Importantly, in all cases the resonance remains within the operating band of the on-body reader antenna (1–2 GHz). This indicates that even significant environmental variations (e.g., due to changes in water/fat content) do not push the resonance out of the operational range. Moreover, such permittivity changes typically occur gradually. Therefore, as long as consecutive measurements are taken within sufficiently short intervals, resonance evolution due to environmental variations will appear

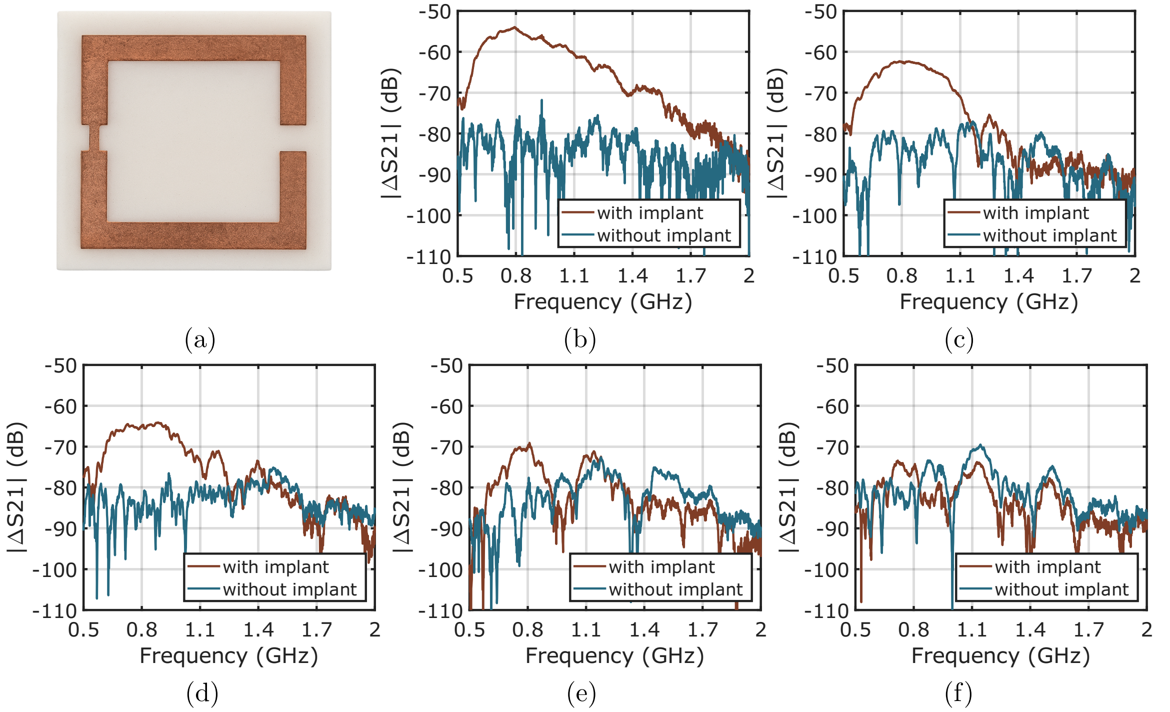

Figure S6: Calibrated transmission coefficient at various implant depths: (a) The non-biodegradable implant antenna used in those measurements, (b) 25 mm, (c) 35 mm, (d) 45 mm, (e) 55 mm, and (f) 65 mm.

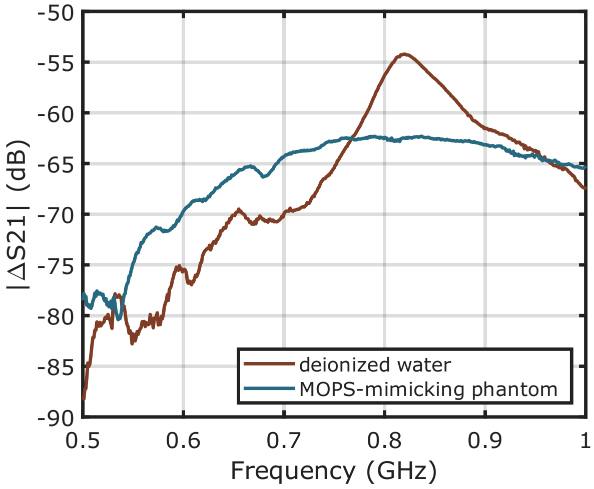

Figure S7: Calibrated transmission coefficients when the implant is placed in either deionized water or a MOPS-mimicking phantom, whose electrical properties are provided in Figure S4. The implant depth is fixed at 35 mm.

smooth. In contrast, degradation of the implant (i.e., structural discontinuity) produces a sudden and sharp change in the spectral response (see Figure 10 of the manuscript), which can be unambiguously detected.

It is also worth noting that the permittivity range $\epsilon_r = 20$–80 encompasses most biological tissues and fluids in the 1–2 GHz range, excluding primarily bone and fat (reference: IT'IS database, https://itis.swiss/virtual-population/tissue-properties/database/dielectric-properties). Hence, the implant remains trackable in physiologically relevant environments. Note that the maximum sensing

depth will be affected by the permittivity and conductivity changes of the immediate tissue.

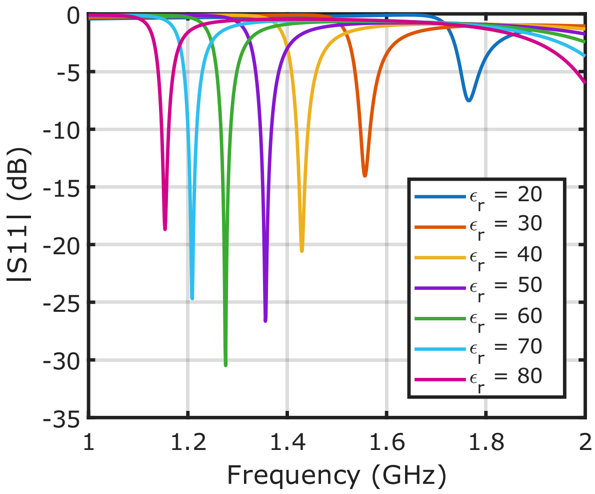

Figure S8: The effect of the permittivity of the media on the resonant frequency of the implant.

The detection process is based on analyzing the calibrated S21 (S21) response of the reader–implant link across 1–2 GHz. The following procedure is employed:

- At each time instant, the maximum —S21— within the band is identified.
- In the intact (non-degraded) state, the spectral maximum remains at the noise floor.
- Degradation is detected once the maximum exceeds a threshold of 10 dB relative to the baseline noise level.

This criterion ensures reliable identification of sudden resonance emergence associated with implant degradation.
