# Supplementary material for: Wireless in-body sensing through genetically engineered bacteria
Source: Nat Commun. 2025 Nov 25;16:10432. doi: 10.1038/s41467-025-65416-5 (PMC12647575; doi:10.1038/s41467-025-65416-5)
Supplement: Supplementary file 2 — Description of Additional Supplementary Files [file 41467_2025_65416_MOESM2_ESM.pdf]

Description of Additional Supplementary Files

Supplementary Data 1- Table 1: Gene sequences used in the study (from 5’ to 3’)
