# Supplementary material for: Wireless in-body sensing through genetically engineered bacteria
Source: Nat Commun. 2025 Nov 25;16:10432. doi: 10.1038/s41467-025-65416-5 (PMC12647575; doi:10.1038/s41467-025-65416-5)
Supplement: Supplementary file 3 — Supplementary Data 1 [file 41467_2025_65416_MOESM3_ESM.pdf]

Table 1: Gene sequences used in the study (from 5' to 3')

Name of the gene	Sequence
ccmA-H	ATGGGTATGCTTGAAGCCAGAGAGTTACTTTGTGAGCGGGATGAACGAAC<br>CTTATTTAGTGGCTTGTCATTTACGCTGAACGCAGGAGAGTGGGTACAAA<br>TCACCGGTAGCAACGGCGCGGGGAAGACAACGCTTCTCCGTTTGCTGACG<br>GGGTTGTCTCGCCCTGACGCAGGCGAGGTTCTCTGGCAAGGGCAGCCCTT<br>GCATCAGGTACGCGACAGCTACCATCAAAACCTGTTATGGATAGGCCATC<br>AGCCGGGGATCAAAACCCGGCTGACGGCGTTAGAAAATCTGCACTTTTAT<br>CATCGCGATGGCGATACCGCACAATGTCTGGAAGCCCTGGCGCAGGCCGG<br>GCTTGCCGGATTCGAAGATATTCCTGTAAATCAGCTCTCGGCCGGGCAAC<br>AACGCCGCGTCGCTTTAGCGCGTCTGTGGCTGACCCGTGCCACGTTATGG<br>ATCCTCGACGAGCCTTTTACCGCGATTGACGTTAACGGTGTCGATCGTCT<br>GACCCAGCGTATGGCGCAGCATACGGAGCAGGGGGGGATTGTGATTCTGA<br>CTACCCACCAGCCGCTCAACGTTGCTGAAAGTAAAATTCGCCGCATTTCA<br>CTGACGCAAACGAGGGCCGCATGAATGATGTTCTGGCGCATTTTCCGTCT<br>TGAGCTGCGTGTAGCGTTTCGCCATAGCGCCGAAATCGCCAACCCGCTGT<br>GGTTCTTCCTGATTGTAATTACCCTTTTTCCGCTCAGTATCGGTCCGGAG<br>CCGCAACTGCTGGCGCGTATTGCACCGGGCATTATCTGGGTTGCTGCGCT<br>GCTTTCATCCTTGCTGGCGCTGGAACGACTGTTCCGTGACGATTTGCAGG<br>ACGGCAGTCTTGAACAATTGATGTTGTTGCCGTTACCCTTGCCCGCCGTT<br>GTGCTGGCGAAGGTGATGGCGCACTGGATGGTAACCGGTCTGCCGTTACT<br>CATCCTTTCGCCACTGGTAGCAATGCTACTGGGAATGGATGTTTATGGCT<br>GGCAAGTGATGGCGCTGACGCTGCTGCTGGGAACGCCTACGCTTGGCTTT<br>CTCGGTGCACCGGGCGTGGCGCTGACAGTGGGACTTAAGCGCGGTGGTGT<br>GCTGCTCAGCATACTGGTGTTACCGCTGACTATCCCATTACTCATCTTTG<br>CCACCGCCGCGATGGACGCGGCTTCTATGCATTTGCCCGTTGACGGGTAT<br>CTGGCAATTTTAGGCGCGTTGCTGGCAGGCACCGCGACATTAAGTCCTTT<br>TGCGACGGCGGCAGCGTTACGAATCAGCATTCAATAAATGTGGAAAACAC<br>TGCATCAACTGGCGATCCCACCACGGCTGTATCAAATCTGTGGCTGGTTT<br>ATACCGTGGCTGGCAATTGCCAGTGTGGTCGTGCTTACCGTCGGCTGGAT<br>CTGGGGATTCGGCTTTGCTCCGGCTGATTATCAGCAGGGAAATAGCTACC<br>GCATTATCTACCTGCATGTGCCTGCGGCGATCTGGTCGATGGGCATTTAT<br>GCATCAATGGCAGTGGCAGCGTTTATTGGCCTTGTCTGGCAGATGAAAAT<br>GGCCAACCTGGCGGTGGCGGCGATGGCCCCCATTGGTGCCGTGTTTACCT<br>TTATTGCCCTGGTTACCGGCTCTGCATGGGGAAAACCGATGTGGGGCACC<br>TGGTGGGTATGGGATGCACGTCTGACTTCTGAACTGGTGCTGCTGTTTTT<br>GTATGTGGGTGTGATTGCCCTGTGGCACGCCTTCGACGACCGCCGTCTGG

CGGGCCGTGCGGCAGGTATCCTGGTGCTGATTGGCGTGGTGAATCTGCCG

ATTATTCATTACTCCGTGGAGTGGTGGAACACCCTGCATCAGGGATCAAC

GCGGATGCAGCAAAGTATCGATCCGGCGATGCGTTCGCCGCTGCGCTGGT

CGATTTTTGGCTTCCTGCTCCTGTCTGCCACGCTGACGCTGATGCGGATG

CGTAATTTGATTTTGCTGATGGAAAAACGCCGTCCGTGGGTGAGTGAACT

GATACTGAAAAGAGGCCGTAAATGAATGACCCCTGCATTTGCTTCCTGGA

ATGAATTTTTCGCAATGGGCGGTTACGCCTTTTTTGTCTGGCTGGCGGTG

GTGATGACCGTTATTCCGCTGGTGGTTTTGGTCGTGCACTCGGTGATGCA

ACATCGCGCAATTCTGCGTGGCGTGGCGCAACAGCGGGCGCGTGAGGCGC

GTTTACGTGCTGCGCAACAGCAGGAGGCTGCATGAATGAATATTCGCCGT

AAAAACCGCTTGTGGATTGCCTGTGCCGTGTTGGCAGGGCTGGCGCTGAC

TATCGGTCTGGTGCTATATGCGCTGCGCTCGAATATCGATCTCTTTTATA

CGCCGGGGGAAATTCTCTACGGCAAGCGTGAAACTCAGCAAATGCCGGAA

GTCGGTCAGCGTCTGCGCGTTGGCGGGATGGTGATGCCGGGTAGTGTGCA

GCGCGATCCCAATTCGCTGAAAGTGACCTTCACCATTTACGATGCTGAAG

GCTCAGTGGATGTCTCTTACGAAGGCATTTTGCCGGATCTGTTCCGTGAA

GGGCAGGGCGTTGTGGTGCAGGGCGAACTGGAAAAAGGCAATCATATCCT

CGCGAAAGAAGTGCTGGCGAAACACGATGAAAACTATACGCCGCCAGAAG

TTGAGAAAGCGATGGAAGCTAACCACCGTCGCCCGGCGAGTGTTTATAAG

GACCCAGCATCATGAATGATGCCAGAAATTGGTAACGGACTGCTGTGCCT

GGCGCTGGGAATTGCGCTGCTGCTGTCCGTGTATCCGCTATGGGGCGTGG

CGCGCGGAGATGCGCGCATGATGGCGTCTTCCCGCTTGTTTGCCTGGCTG

CTGTTTATGTCTGTGGCTGGCGCATTTCTGGTGCTGGTCAATGCTTTCGT

GGTCAACGACTTCACCGTCACCTATGTTGCCAGCAACTCCAATACCCAGC

TTCCGGTGTGGTATCGCGTGGCGGCTACCTGGGGCGCGCATGAAGGCTCG

CTACTGCTGTGGGTGCTGCTGATGAGCGGCTGGACCTTTGCGGTGGCGAT

TTTTAGTCAGCGTATTCCGCTGGATATTGTGGCCCGTGTACTGGCGATAA

TGGGGATGGTCAGTGTCGGCTTTTTGCTGTTCATTCTCTTTACCTCTAAC

CCGTTCTCTCGCACGTTGCCGAACTTCCCGATTGAAGGTCGCGATCTTAA

CCCGCTATTGCAGGATCCGGGGCTGATCTTCCATCCGCCTCTGCTTTATA

TGGGGTACGTGGGTTTCTCGGTGGCGTTTGCTTTTGCCATTGCTTCTTTG

CTGAGCGGGCGTCTGGACAGCACTTATGCGCGTTTTACTCGTCCGTGGAC

GCTGGCAGCGTGGATCTTCCTGACGCTCGGCATCGTGCTCGGTTCCGCAT

GGGCCTATTACGAACTCGGCTGGGGTGGCTGGTGGTTCTGGGATCCGGTA

GAAAACGCCTCGTTTATGCCGTGGCTGGTGGGGACTGCGCTGATGCACTC

ACTGGCGGTCACTGAACAACGCGCCAGCTTCAAAGCGTGGACATTACTGC

TGGCAATCAGTGCCTTCTCGTTGTGTCTGTTGGGGACTTTCCTCGTGCGT

TCCGGCGTGCTGGTATCGGTACACGCGTTTGCGTCTGATCCGGCGCGCGG

TATGTTTATCCTCGCCTTTATGGTGCTGGTGATTGGCGGTTCGCTGCTGC
TGTTTGCCGCGCGTGGACACAAAGTTCGCTCACGCGTAAACAATGCGCTG
TGGTCGCGGGAATCTTTGCTGTTAGCGAACAATGTTTTGCTGGTCGCTGC
GATGCTGGTGGTGTTGCTGGGGACGCTGCTGCCGTTGGTGCATAAGCAAC
TGGGACTGGGCAGTATTTCGATTGGCGAACCGTTCTTCAACACCATGTTT
ACCTGGCTGATGGTGCCGTTTGCGCTACTGCTTGGTGTCGGTCCTCTGGT
GCGCTGGGGGCGGGATCGCCCGCGTAAGATCCGCAATTTATTGATTATCG
CCTTCATCTCTACGCTGGTGCTGTCGCTGCTGTTGCCGTGGCTGTTCGAA
AGCAAAGTTGTGGCGATGACGGTGCTCGGCCTGGCAATGGCCTGCTGGAT
TGCGGTGCTGGCAATTGCGGAAGCTGCGCTACGTATTTCACGCGGCACGA
AAACCACCTTCAGTTATTGGGGGATGGTGGCGGCTCACCTTGGGCTGGCA
GTGACAATTGTTGGCATTGCCTTTAGCCAGAACTATAGCGTTGAGCGTGA
TGTGCGCATGAAGTCCGGCGATAGCGTCGATATTCATGAATATCGCTTCA
CCTTCCGTGATGTCAAAGAGGTGACTGGCCCGAACTGGCGTGGCGGTGTG
GCGACTATCGGCGTAACGCGCGATGGCAAGCCGGAAACGGTGCTGTATGC
GGAAAAACGTTATTACAACACTGCCGGGTCGATGATGACCGAAGCGGCAA
TTGACGGCGGCATCACGCGTGACCTGTACGCGGCCCTCGGTGAAGAGCTG
GAAAACGGCGCGTGGGCCGTGCGTCTTTACTACAAACCATTTGTTCGCTG
GATTTGGGCGGGCGGGCTGATGATGGCGTTGGGCGGACTGCTGTGTCTGT
TTGATCCTCGCTATCGTAAGCGCGTGAGTCCGCAAAAAACTGCGCCGGAG
GCCGTATGAATGAAGCGCAAAGTATTGTTAATTCCGTTGATTATCTTCCT
GGCGATTGCCGCGGCGCTGCTGTGGCAGCTGGCGCGTAATGCCGAAGGGG
ATGATCCGACCAATCTGGAATCGGCGCTCATTGGCAAGCCTGTGCCGAAG
TTTCGTCTCGAATCACTGGACAATCCGGGGCAGTTTTATCAGGCGGATGT
GCTGACTCAGGGCAAACCAGTACTGCTTAACGTCTGGGCGACCTGGTGTC
CGACCTGCCGTGCGGAACATCAATATCTGAATCAGCTTTCTGCGCAGGGC
ATCCGCGTGGTCGGCATGAACTATAAAGACGATCGCCAGAAGGCAATCAG
CTGGCTGAAAGAGCTGGGCAATCCTTACGCGCTAAGCCTGTTTGATGGCG
ATGGCATGTTAGGGCTGGATCTCGGTGTCTATGGCGCGCCAGAAACGTTT
CTTATTGACGGCAACGGCATCATTCGCTATCGCCATGCGGGCGATCTGAA
TCCTCGCGTCTGGGAAGAAGAGATCAAGCCGCTGTGGGAGAAATACAGTA
AGGAGGCCGCACAATGAATGAGGTTTTTATTGGGCGTGCTGATGCTGATG
ATCTCCGGCTCAGCGCTGGCGACCATCGATGTGTTGCAGTTTAAAGATGA
AGCACAGGAACAACAGTTCCGTCAGCTCACTGAAGAACTGCGCTGCCCGA
AATGCCAGAACAACAGCATTGCCGATTCCAACTCGATGATTGCCACCGAC
CTGCGTCAGAAAGTGTATGAACTGATGCAGGAAGGTAAAAGTAAAAAAGA
GATTGTCGATTATATGGTGGCGCGTTACGGCAACTTCGTCACTTACGATC
CGCCGTTAACGCCGCTGACCGTGCTGCTGTGGGTGCTGCCAGTAGTGGCT

	ATTGGCATTGGCGGTTGGGTCATATACGCCCGCTCGCGGCGTCGGGTACG<br>CGTGGTGCCGGAAGCGTTTCCTGAACAAAGCGTGCCGGAAGGTAAGCGTG<br>CCGGATATGTTGTTTATCTGCCGGGTATTGTGGTGGCGTTAATTGTGGCT<br>GGCGTCAGCTACTACCAGACTGGCAATTATCAGCAGGTGAAAATCTGGCA<br>GCAGGCCACGGCACAGGCTCCGGCGTTGCTGGACAGGGCGCTGGATCCGA<br>AAGCCGATCCGCTCAACGAAGAAGAGATGTCGCGTCTTGCGCTGGGGATG<br>CGTACTCAACTGCAAAAAAATCCGGGAGATATAGAAGGCTGGATTATGTT<br>GGGCCGCGTTGGCATGGCGCTGGGTAACGCCAGTATCGCCACCGATGCAT<br>ACGCCACTGCGTATCGCCTCGATCCGAAAAACAGTGATGCTGCACTGGGA<br>TACGCTGAAGCGTTGACTCGTTCATCTGATCCCAACGACAACCGCCTCGG<br>CGGTGAACTGCTACGTCAGCTGGTGAGAACGGACCATAGCAATATCCGTG<br>TGCTAAGCATGTATGCGTTTAATGCCTTTGAGCAGCAGCGATTTGGCGAA<br>GCCGTTGCCGCGTGGGAGATGATGTTGAAACTCTTACCTGCCAACGATAC<br>TCGCCGTGCGGTGATTGAACGTAGTATCGCGCAGGCGATGCAACATTTGT<br>CGCCGCAGGAGAGTAAATAA

**Regulatory DNA part**	**Sequence**
mProD promoter	tctagaTTTACAGCTAGCTCAGTCCTAGGTATAATGCTAGCtactagag
Ribosome binding site (RBS)	AAAGAGGAGAAA
rrnB T1 terminator	GGCATCAAATAAAACGAAAGGCTCAGTCGAAAGACTGGGCCTTTCGTTTT<br>ATCTGTTGTTTGTCGGTGAACGCTCTCCTGAGTAGGACAAATCCGCCGCC<br>CTAGA
T7 Terminator	CTAGCATAACCCCTTGGGGCCTCTAAACGGGTCTTGAGGGGTTTTTTG
Kan promoter	gctggggcgccctctggtaaggttgggaagccctgcaa
Kan resistance gene	attgaacaagatggattgcacgcaggttctccggccgcttgggtggagag<br>gctattcggctatgactgggcacaacagacaatcggctgctctgatgccg<br>ccgtgttccggctgtcagcgcaggggcgcccggttctttttgtcaagacc<br>gacctgtccggtgccctgaatgaactgcaggacgaggcagcgcggctatc<br>gtggctggccacgacgggcgttccttgcgcagctgtgctcgacgttgtca<br>ctgaagcgggaagggactggctgctattgggcgaagtgccggggcaggat<br>ctcctgtcatctcaccttgctcctgccgagaaagtatccatcatggctga<br>tgcaatgcggcggctgcatacgcttgatccggctacctgcccattcgacc<br>accaagcgaaacatcgcatcgagcgagcacgtactcggatggaagccggt<br>cttgtcgatcaggatgatctggacgaagagcatcaggggctcgcgccagc<br>cgaactgttcgccaggctcaaggcgcgcatgcccgacggcgaggatctcg<br>tcgtgacccatggcgatgcctgcttgccgaatatcatggtggaaaatggc<br>cgcttttctggattcatcgactgtggccggctgggtgtggcggaccgcta<br>tcaggacatagcgttggctacccgtgatattgctgaagagcttggcggcg

	aatgggctgaccgcttcctcgtgctttacggtatcgccgctcccgattcg<br>cagcgcatcgccttctatcgccttcttgacgagttcttctga
lambda T0 terminator	gactcctgttgatagatccagtaatgacctcagaactccatctggatttg<br>ttcagaacgctcggttgccgccgggcgttttttattggtgagaat
Origin of replication	gccttactgggtgcattagccagtctgaatgacctgtcacgggataatcc<br>gaagtggtcagactggaaaatcagagggcaggaactgctgaacagcaaaa<br>agtcagatagcaccacatagcagacccgccataaaacgccctgagaagcc<br>cgtgacgggcttttcttgtattatgggtagtttccttgcatgaatccata<br>aaaggcgcctgtagtgccatttacccccattcactgccagagccgtgagc<br>gcagcgaactgaatgtcacgaaaaagacagcgactcaggtgcctgatggt<br>cggagacaaaaggaatattcagcgatttgcccgagcttgcgagggtgcta<br>cttaagcctttagggttttaaggtctgttttgtagaggagcaaacagcgt<br>ttgcgacatccttttgtaatactgcggaactgactaaagtagtgagttat<br>acacagggctgggatctattctttttatctttttttattctttctttatt<br>ctataaattataaccacttgaatataaacaaaaaaaacacacaaaggtct<br>agcggaatttacagagggtctagcagaatttacaagttttccagcaaagg<br>tctagcagaatttacagatacccacaactcaaaggaaaaggactagtaat<br>tatcattgactagcccatctcaattggtatagtgattaaaatcacctaga<br>ccaattgagatgtatgtctgaattagttgttttcaaagcaaatgaactag<br>cgattagtcgctatgacttaacggagcatgaaaccaagctaattttatgc<br>tgtgtggcactactcaaccccacgattgaaaaccctacaaggaaagaacg<br>gacggtatcgttcacttataaccaatacgctcagatgatgaacatcagta<br>gggaaaatgcttatggtgtattagctaaagcaaccagagagctgatgacg<br>agaactgtggaaatcaggaatcctttggttaaaggctttgagattttcca<br>gtggacaaactatgccaagttctcaagcgaaaaattagaattagtttta<br>gtgaagagatattgccttatcttttccagttaaaaaaattcataaaatat<br>aatctggaacatgttaagtcttttgaaaacaaatactctatgaggattta<br>tgagtggttattaaaagaactaacacaaaagaaaactcacaaggcaaata<br>tagagattagccttgatgaatttaagttcatgttaatgcttgaaaataac<br>taccatgagtttaaaaggcttaaccaatgggttttgaaaccaataagtaa<br>agatttaaacacttacagcaatatgaaattggtggttgataagcgaggcc<br>gcccgactgatacgttgattttccaagttgaactagatagacaaatggat<br>ctcgtaaccgaacttgagaacaaccagataaaaatgaatggtgacaaaat<br>accaacaaccattacatcagattcctacctacgtaacggactaagaaaaa<br>cactacacgatgctttaactgcaaaaattcagctcaccagttttgaggca<br>aaatttttgagtgacatgcaaagtaagcatgatctcaatggttcgttctc<br>atggctcacgcaaaaacaacgaaccacactagagaacatactggctaaat<br>acggaaggatctgaggttcttatggctcttgtatctatcagtgaagcatc

	aagactaacaaacaaaagtagaacaactgttcaccgttagatatcaaagg<br>gaaaactgtccatatgcacagatgaaaacggtgtaaaaaagatagataca<br>tcagagcttttacgagtttttggtgcatttaaagctgttcaccatgaaca<br>gatcgacaatgtaacagatgaacagcatgtaacacctaatagaacaggtg<br>aaaccagtaaaacaaagcaactagaacatgaaattgaacacctgagacaa<br>cttgttacagctcaacagtcacacatagacagcctgaaacaggcgatgct<br>gcttatcgaatcaaagctgccgacaacacgggagccagtgacgcctcccg<br>tggggaaaaaatcatggcaattctggaagaaatagcgctttcagccggca<br>aacctgaagccggatctgcgattctgataacaaactagcaacaccagaac<br>agc
